# Phase-controlled coherent photons for the quantum correlations in a delayed-choice quantum eraser scheme

**DOI:** 10.1038/s41598-024-52125-0

**Published:** 2024-01-19

**Authors:** Byoung S. Ham

**Affiliations:** 1 School of Electrical Engineering and Computer Science, Gwangju Institute of Science and Technology, 123 Chumdangwagi-ro, Buk-gu, Gwangju, 61005 South Korea; 2 Qu-Lidar, 123 Chumdangwagi-ro, Buk-gu, Gwangju, 61005 South Korea

**Keywords:** Physics, Quantum mechanics

## Abstract

The delayed-choice quantum eraser has been intensively studied for the wave-particle duality of a single photon in an interferometric system over the last decades. Super-resolution has been studied over decades for quantum sensing to overcome the standard quantum limit. For the super-resolution, either quantum features of higher-order entangled photon pairs or classical features of phase-controlled coherent photons have been successfully demonstrated. Here, a method of classically excited super-resolution is presented for the phase-controlled coherent photons in a quarter-wave plate-modified quantum eraser scheme. To support the underlying physics of the super-resolution, nonlocal correlation is also presented with an additional frequency-polarization basis control via selective product-basis measurements.

## Introduction

Quantum mechanics has been developed based on the wave-particle duality^1^ of a single particle over the last century, resulting in various quantum technologies in computing^2–4^, communications^5–7^, and sensing areas^8–10^. In a single photon’s self-interference^11^, quantum superposition between orthonormal bases of the single photon plays an essential role^12–15^. The wave-particle duality originated in quantum superposition must be exclusive in their natures according to the Copenhagen interpretation^16–18^. In that sense, the delayed-choice quantum eraser^12–15,19^ can be understood as an ad-hoc quantum superposition of a measured photon through a dynamic window of a polarizer^19^. Due to the exclusive nature of the wave-particle duality, the violation of the cause-effect relation in the quantum eraser can also be understood for the superposition of many waves resulting in the bandwidth-limited effective coherence, where no quantum feature exists beyond the ensemble coherence^19^.

Quantum entanglement is between two or more individual photons (particles) whose presumably assumed fixed phase relation between paired photons does not violate quantum mechanics^20^. A typical entangled photon pair is obtained by a spontaneous parametric down-conversion (SPDC) process of the second-order nonlinear optics^21^. Because the wave mixing process of SPDC is coherent, a phase matching among the pump and two sibling photons is an inherent birth condition^20–23^. In that sense, an assumption of a specific phase relation between the entangled photons is not absurd. Recently, such an understanding has been presented for the Hong-Ou-Mandel (HOM) effect^24,25^ and Franson-type nonlocal correlation^26^ using the wave nature. Experimental demonstrations have also been conducted in trapped ions for a $\uppi /2$ phase difference^27^. A complete analytical solution of the phase relation between entangled photons shows the same $\uppi /2$ for the HOM effect^25^. For the nonlocal correlation between entangled photons, a coincidence measurement process for a particular product-basis selection is an essential condition^5,22,26,28^. Thus, the quantum feature should be understood differently.

**Figure 1:**
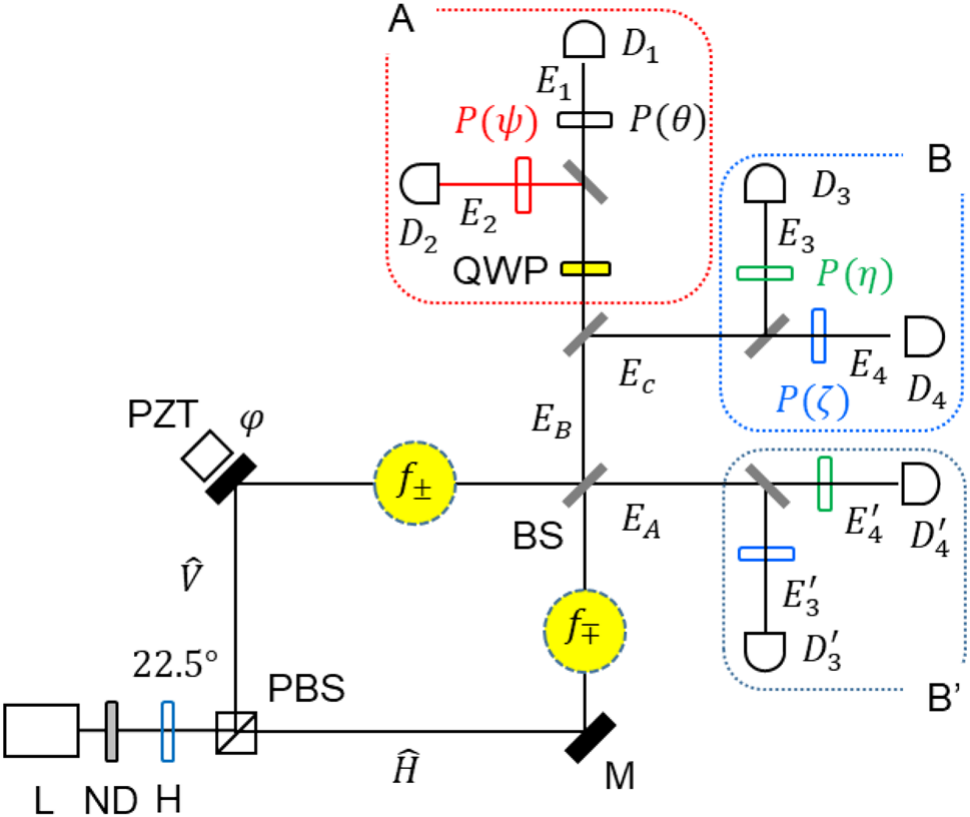
Schematic of coherence manipulations of coherent photons for a photonic de Broglie wave in a delayed-choice quantum eraser. L: laser, ND: neutral density filter, H: half-wave plate, PBS: polarizing beam splitter, PZT: piezo-electric transducer, M: mirror, BS: nonpolarizing 50/50 beam splitter, QWP: quarter-wave plate, P: polarizer, D: single photon detector, H: horizon polarization, V: vertical polarization. ${f}_{\pm }$ and ${f}_{\mp }$ are generated by a pair of acousto-optic modulators. All rotation angles of Ps are independent of each other, satisfying local realism.

**Figure 2:**
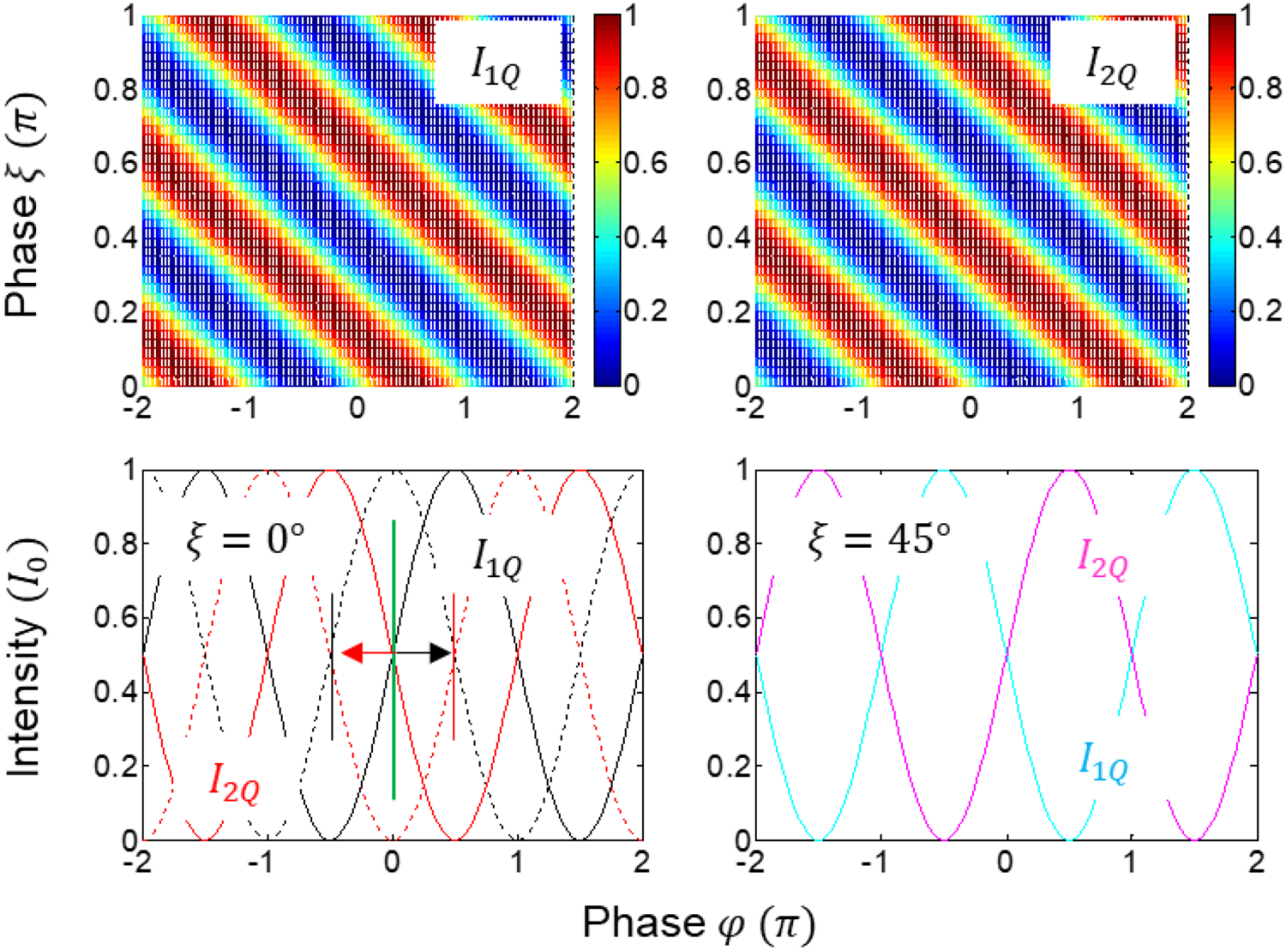
Numerical calculations of the QWP-induced fringe shift in the quantum eraser. (lower left panel) black and red dotted curves are for Eqs. (7) and (8), respectively. $\uptheta =\uppsi =\upeta =\upzeta =\uppi /4$.

Here, completely different methods of super-resolution^29–39^ and nonlocal correlation^40–42^ are presented using phase and frequency-controlled coherent photons, respectively, where the super-resolution is to overcome the standard quantum limit (SQL) toward the Heisenberg limit, as shown by photonic de Broglie wave (PBWs)^29–32^, N00N states^35–39^, or squeezed state^39^. Classically excited super-resolution has also been observed for the Heisenberg limit in quantum sensing using phase-controlled coherent photons^33,34^ via the projection method^37^ in a non-interferometric system. For the coherence manipulations of the phase control for super-resolution, a quarter-wave plate (QWP) is inserted before the polarizer in the quantum eraser scheme of Fig. 1^19^. The QWP-caused phase shift between orthogonal polarization bases of coherent photon results in fringe shifts between with and without QWP-based quantum erasers (see Fig. 2)^43^. For the coherently excited nonlocal correlations, a pair of acousto-optic modulators (AOMs) are used to induce the polarization-frequency correlation for post-determined coincidence measurements^25^. For the excitation of the coherently excited nonlocal quantum features between quantum erasers, a selective measurement technique plays a key role for the inseparable intensity products, where DC components are blocked.

## Result

### Phase control of coherent photons in a quantum eraser scheme

Figure 1 shows the schematic of a modified delayed-choice quantum eraser using phase controls of Poisson-distributed photons with, firstly, QWP only for the PBW-like super-resolution and, secondly, for the nonlocal quantum feature with an additional pair of AOMs. The photon number entering the noninterfering Mach–Zehnder interferometer (MZI) is post-determined by coincidence detection^19^. By the polarizing beam splitter (PBS), distinguishable photon characteristics are provided inside the MZI. For the polarization-basis manipulations of the distinguishable photons, a polarizer (P) is added before each single-photon detector ${{\text{D}}}_{j}$(j = 1–4), resulting in the quantum eraser^19^. Thus, the particle nature of the photon is known retrospectively to turn out to be the wave nature in the first-order intensity correlation^12–15^. This is a generally accepted quantum feature of the cause-effect violation, where the physical distances between PBS and Ps are kept beyond the light cone^13,15^. This condition is also equivalently accepted for the violation of local realism in the second-order intensity correlation^28,42^.

For the first task of the classically excited super-resolution overcoming the diffraction limit or SQL in phase resolution, a QWP is inserted in one of the output ports of MZI (see block A). As a result, an opposite fringe shift occurs between quantum erasers with and without QWP, i.e., between blocks A and B in Fig. 1 (see Fig. 2). Although, such a phase-controlled coherent photon-induced fringe shifts in the first-order intensity correlations have been observed in non-interferometric systems for the PBW-like super-resolution^33,34^, the scheme of Fig. 1 is unprecedented based on MZI of the quantum eraser. The physics of this fringe shift is the QWP-induced phase shift between orthogonal polarization bases of a single photon (see “Analysis”)^44^. Thus, the second-order intensity correlation between the quantum erasers from either the same side (between blocks A and B) or opposite sides (between blocks A and B’) needs to be analyzed for the direct proof of the super-resolution proportional to the intensity order (see “Analysis”). Very recently, experimental demonstrations of the classically excited super-resolution have been conducted^43^. Here, the super-resolution satisfies the Heisenberg limit, where super-sensitivity is a different research field^37,38^. Secondly, intensity products between two quantum erasers with and without QWP are post-controlled to discard (or choose) particular product bases, as shown for the Hong-Ou-Mandel effect^25^ and Franson-type nonlocal correlation^26,28^. For this, a gated heterodyne detection technique^45^ is adopted for the AOM-induced frequency-polarization correlation of paired photons at ${f}_{\pm }={f}_{0}\pm \delta f$, where the quantum state inside the MZI is represented by $|\Psi \rangle ={|f\rangle }_{\mp }|H\rangle +{e}^{i\varphi }{|f\rangle }_{\pm }|V\rangle$. The coherence solution of the quantum eraser for the first-order intensity correlation has no objection to the quantum approach due to the analytical equality to the classical one^18,46^.

The QWP inserted one output port of MZI in Fig. 1 induces a phase retardation of $\pm\uppi /2$ to the vertical polarization basis of a photon to the horizontal one, where the $\pm$ signs depend on the principle axis’ rotation angles (0, $\uppi /2$) of the QWP^44^. This polarization-dependent phase retardation should directly affect the quantum eraser because the role of the polarizer P is to project orthogonal polarization bases onto the common axis (see Eqs. (3)–(6))^19^. In general, an interferometer is insensitive to the global phase of a photon due to the Born rule, where the measurement is the absolute square of the probability amplitude^17,18^. Interestingly, the phase control of the output photon by QWP directly results in the fringe shift of the quantum eraser (see Fig. 2)^43^. This phenomenon is unprecedented in coherence optics even for phase-controlled super-resolution^32,33^. In quantum mechanics, such a fringe shift of the second-order intensity product not for $\mathrm{\varphi }$ but for $\uptheta$ $(\uppsi ,\upeta ,\mathrm{ or \zeta })$ has been the witness of the nonlocal quantum correlation (see Fig. 4)^47^.

### Analysis 1: PBW-like quantum feature

A coherence approach based on the wave nature of a photon is adopted to analyze Fig. 1 differently from the quantum approach based on quantum operators^20,36–39^. This means that the present “Analysis” is classical. The novel feature of the present method is to result in reduced PBWs eliminating unwanted side effects by split photons on BS^37,38^ of MZI in Fig. 1^43^. For a normal quantum eraser scheme without QWP and AOMs^25^, the amplitudes of output fields from the MZI in Fig. 1 are represented by:

1 $${{\varvec{E}}}_{A}=\frac{i{E}_{0}}{2}\left(\widehat{H}+\widehat{V}{e}^{i\varphi }\right),$$

2 $${{\varvec{E}}}_{B}=\frac{{E}_{0}}{2}\left(\widehat{H}-\widehat{V}{e}^{i\varphi }\right),$$

where ${E}_{0}$ is the amplitude of a single photon from an attenuated laser L. For the present single-photon regime satisfying independent measurement events in statistics, the mean photon number $\langle n\rangle$ is adjusted to be $\langle n\rangle \ll 1$^19,43^. As demonstrated^43^, the input photon number N entering MZI for the higher-order intensity correlation is post-determined by coincidence measurements between quantum erasers^30,32,37,43^. $\widehat{H}$ and $\widehat{V}$ are unit vectors of horizontal and vertical polarization bases of a photon, respectively. Due to the orthogonal bases, Eqs. (1) and (2) result in no $\mathrm{\varphi }$-dependent fringes, resulting in $\langle {I}_{A}\rangle =\langle {I}_{A}\rangle ={I}_{0}/2$^19^. By the rotated polarizers whose rotation angles are from the horizontal axis, Eq. (2) is modified for the following quantum erasers:

3 $${{\varvec{E}}}_{1}=\frac{{E}_{0}}{4}\left(\widehat{H}\cos\theta -\widehat{V}\sin\theta {e}^{i\varphi }\right)\widehat{p},$$

4 $${{\varvec{E}}}_{2}=\frac{-i{E}_{0}}{4}\left(\widehat{H}\cos\psi +\widehat{V}\sin\psi {e}^{i\varphi }\right)\widehat{p},$$

5 $${{\varvec{E}}}_{3}=\frac{-{E}_{0}}{4}\left(\widehat{H}\cos\eta -\widehat{V}\sin\eta {e}^{i\varphi }\right)\widehat{p},$$

6 $${{\varvec{E}}}_{4}=\frac{-i{E}_{0}}{4}\left(\widehat{H}\cos\zeta +\widehat{V}\sin\zeta {e}^{i\varphi }\right)\widehat{p},$$

where $\widehat{p}$ is the axis of the polarizers. In Eqs. (3)–(6), the insertion of meaningless $\widehat{H}$ and $\widehat{V}$ is just to indicate the photon’s origin to understand the role of Ps, where only $\widehat{H}$ is reversed by the BS, as shown in the mirror image. For the block B’, ${\mathbf{E}}_{3}^{{{\prime}}}=\frac{{E}_{0}}{4}\left(\widehat{H}\cos\eta -\widehat{V}\sin\eta {e}^{i\varphi }\right)\widehat{p}$ and ${\mathbf{E}}_{4}^{{{\prime}}}=\frac{i{E}_{0}}{4}\left(\widehat{H}\cos\zeta +\widehat{V}\sin\zeta {e}^{i\varphi }\right)\widehat{p}$ are resulted. Due to the no action of the global phase on measurements by Born’s rule, $\langle {I}_{3}^{\prime}\rangle =\langle {I}_{3}\rangle$ and $\langle {I}_{4}^{\prime}\rangle =\langle {I}_{4}\rangle$ are obtained. In other words, the quantum eraser schemes of blocks B and B’ are identical for their fringes.

Thus, the corresponding mean intensities are described as follows for $\upeta =\uptheta$ and $\upzeta =\uppsi$:

7 $$\langle {I}_{1}\rangle =\langle {I}_{3}\rangle =\langle {{\text{I}}}_{3}^{\prime}\rangle =\frac{{I}_{0}}{16}\left(1-\sin2\theta \cos\varphi \right),$$

8 $$\langle {I}_{2}\rangle =\langle {I}_{4}\rangle =\langle {{\text{I}}}_{4}^{\prime}\rangle =\frac{{I}_{0}}{16}\left(1+\sin2\psi \cos\varphi \right).$$

The quantum mystery of the cause-effect relation of the quantum eraser can be found in the ad-hoc basis superposition of a single photon determined by $\widehat{p}$ of the polarizer at the cost of 50% photon loss (see Section A of the Supplementary Materials). The global phase in Eqs. (3)–(6) does not affect the intensity fringe by the Born rule, as shown in Eqs. (7) and (8). For the balanced polarization control of the polarizer at $\uptheta =\uppsi =45^\circ$, $\langle {I}_{1}\rangle =\langle {I}_{3}\rangle =\frac{{I}_{0}}{16}\left(1-\cos\varphi \right)$ and $\langle {I}_{2}\rangle =\langle {I}_{4}\rangle =\frac{{I}_{0}}{16}\left(1+\cos\varphi \right)$ are resulted, as in the usual MZI, where fringes appear at $\mathrm{\varphi }=2\mathrm{n\pi }$ (see Fig. 2). The fringe inversion between Eqs. (7) and (8), is due to the $\uppi$-phase shift between $\widehat{V}$ components. Recently a complete coherence solution of the quantum eraser has been experimentally demonstrated in a single photon regime^19^.

**Figure 3:**
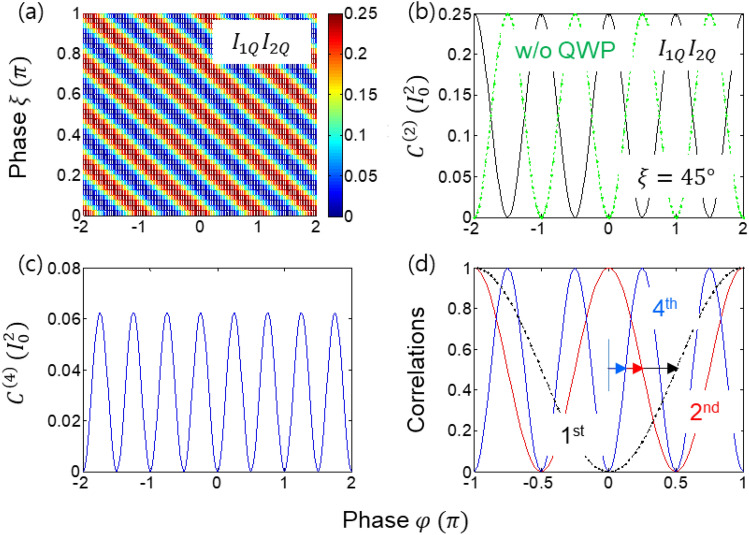
Numerical calculations for the phase quantization of the intensity product. (middle panel) ${C}^{(2)}={C}_{1Q2Q}^{\xi }$. (right panel) ${C}^{(4)}$ is the ${C}^{\left(2\right)}(Black){C}^{\left(2\right)}(green)$ in the middle panel.

Now, a modified quantum eraser of Fig. 1 is analyzed with an inserted QWP in one output port of MZI, resulting in an opposite fringe shift between quantum erasers in that port (see Fig. 2). This QWP-induced fringe shift is the origin of the super-resolution for the higher-order intensity products, resulting in the PBW-like quantum feature (see Fig. 3)^43^. In coherence optics, a QWP whose slow-axis is horizontal ($0^\circ$) induces a phase gain of $\uppi /2$ to the $\widehat{V}$ component^44^. Thus, Eqs. (3) and (4) are written for the slow-axis horizontal (SA-H) QWP as follows:

9 $${{\varvec{E}}}_{1Q}=\frac{{E}_{0}}{4}\left(\widehat{H}\cos\theta -i\widehat{V}\sin\theta {e}^{i\varphi }\right)\widehat{p},$$

10 $${{\varvec{E}}}_{2Q}=\frac{-i{E}_{0}}{4}\left(\widehat{H}\cos\psi +i\widehat{V}\sin\psi {e}^{i\varphi }\right)\widehat{p}.$$

The corresponding intensities are given by:

11 $$\langle {I}_{1Q}\rangle =\frac{{I}_{0}}{16}\left(1+\sin2\theta \sin\varphi \right),$$

12 $$\langle {I}_{2Q}\rangle =\frac{{I}_{0}}{16}\left(1-\sin2\psi \sin\varphi \right).$$

For $\uptheta =\uppsi =45^\circ$, Eqs. (11) and (12) show $\mp\uppi /2$ phase-shifted fringes with respect to Eqs. (7) and (8), respectively (see the arrows in the lower left panel of Fig. 2). For the QWP whose slow-axis is vertical ($90^\circ$), the fringes in Eqs. (11) and (12) are revered due to the sign reversal in $\widehat{V}$ component^43^. Similarly, such a fringe shift for the first-order intensity correlation has been observed in a non-interferometric system via phase control of coherent photons for the same super-resolution overcoming SQL^33,34^.

For a generalized case with an arbitrary angle $\upxi$ of the QWP at SA-H, the orthogonal polarization bases are represented by $\widehat{{\text{H}}}\to \widehat{{\text{H}}}{e}^{-i2\xi }$ and $\widehat{{\text{V}}}\to \widehat{{\text{V}}}{e}^{i2\xi }$^35^. Then, Eqs. (9) and (10) are rewritten as:

13 $${\mathbf{E}}_{1Q}^{\xi }=\frac{{E}_{0}}{4}{e}^{-i2\xi }\left(\widehat{H}\cos\theta -i\widehat{V}\sin\theta {e}^{i(\varphi +4\xi )}\right)\widehat{p},$$

14 $${\mathbf{E}}_{2Q}^{\xi }=\frac{-i{E}_{0}}{4}{e}^{-i2\xi }\left(\widehat{H}\cos\theta +i\widehat{V}{e}^{i2\xi }\sin\theta {e}^{i(\varphi +4\xi )}\right)\widehat{p}.$$

Thus, the corresponding intensities are obtained as:

15 $$\langle {I}_{1Q}^{\xi }\rangle =\frac{{I}_{0}}{16}\left(1+\sin2\theta \sin(\varphi +4\xi )\right),$$

16 $$\langle {I}_{2Q}^{\xi }\rangle =\frac{{I}_{0}}{16}\left(1-\sin2\theta \sin(\varphi +4\xi )\right).$$

For $\upxi =0^\circ$, $\langle {I}_{1Q}^{\xi =0}\rangle =\frac{{I}_{0}}{16}\left(1+\sin2\theta \sin\varphi \right)$ and $\langle {I}_{2Q}^{\xi =0}\rangle =\frac{{I}_{0}}{16}\left(1-\sin2\psi \sin\varphi \right)$ confirm Eqs. (11) and (12).

Figure 2 shows the numerical calculations of Eqs. (15) and (16). As shown in the upper panels, the relations between Eqs. (15) and (16) are confirmed for the opposite fringes^43^. In the lower-left panel, Eqs. (11) and (12) are also confirmed for the fringe shifts of $\mp\uppi /2$ with respect to the reference of Eqs. (7) and (8), as indicated by colored arrows. For $\upxi =45^\circ$, ${I}_{1Q}^{\xi =45}={I}_{2Q}^{\xi =0}$ and ${I}_{2Q}^{\xi =45}={I}_{1Q}^{\xi =0}$ are resulted, as shown in the lower right panel. Thus, there is no difference between $\upxi =0^\circ$ and $\upxi =90^\circ$ for the QWP-modified quantum eraser.

The direct intensity product between Eqs. (15) and (16) for doubly-bunched coherent photons (N = 2) is described as for $\upxi =0$ and $\uptheta =\uppsi =\uppi /4$ (see the upper panels of Fig. 3):

17 $$\langle {{\text{C}}}_{1Q2Q}^{\xi =0}(0)\rangle =\frac{{I}_{0}^{2}}{64}{\cos}^{2}\varphi ,$$

Here, a common factor $\sqrt{2}$ is multiplied to Eqs. (9) and (10) for the two-photon condition of the doubly bunched input-photon case for the coincidence detection. Thus, the number of fringes is doubled as the photon number is doubled in Eq. (17). This result shows the same super-resolution observed by PBWs^29–32^ and phase-controlled coherent photons^33,34^. Unlike the entangled photon-based super-resolution^37,38^, the fringe visibility is nearly perfect due to the complete elimination of the split photons on BS^43^. Without QWP, no PBW-like super-resolution occurs, unless cross-intensity products are measured between MZI output ports (see Section B of the Supplementary Materials). Thus, Eq. (17) shows the super-resolution satisfying Heisenberg limit for N = 2.

The fourth-order intensity product between all quantum erasers of Eqs. (7), (8), and (17) for $\uptheta =\uppsi =\upeta =\upzeta =\uppi /4$ is as follows:

18 $$\langle {{\text{C}}}^{\left(4\right)}(0)\rangle =\frac{{I}_{0}^{4}}{256}{\sin}^{2}\varphi {\cos}^{2}\varphi ,$$

where a common factor 2 is multiplied to Eqs. (9) and (10) for the four-photon condition. Again, Eq. (18) shows the same fringe doubling as Eq. (17), resulting in the quadruple fringes compared to Fig. 2 (see lower left panel of Fig. 3). Obviously, all equations from (1) to (18) are for classical physics. Although, the fringe doubling effects in Eqs. (11), (17), and (18) for N = 1, 2, 4 have been observed for super-resolution using phase-controlled coherent photons^33,34^, the quantum eraser scheme is unprecedented and the related physics is also different (see “Analysis 2: Nonlocal correlation”). The detailed analysis of the classical version limited by the standard quantum limit for Fig. 1 without QWP is discussed in Section C of the Supplementary materials.

Figure 3 shows the numerical calculations of Eqs. (7), (8), (15), (16), and (18) for the super-resolution using phase-controlled coherent photons. Figure 3(a) is for the second-order intensity correlation between Eqs. (15) and (16), showing the fringe doubling compared to Fig. 2. Figure 3b shows a $\uppi /2$ fringe shift between the intensity products with (block A) and without (block B) QWP. Figure 3c is for Eq. (18) for the quadrupled fringes compared to Fig. 2. Finally, Fig. 3d is the PBW-like quantum feature for the N-proportional super-resolution whose resolution follows the Heisenberg’s limit. Thus, the proposed phase-controlled coherent photon-based super-resolution for N = 1–4 is numerically confirmed. For the experimental demonstrations, refer to ref.^43^. Compared with N00N state-based super-resolution, the present method has no side effect caused by split photons for N = 4^37^. The nonlocal quantum feature of this scheme is analyzed below in “Analysis 2: Nonlocal correlation”.

### Analysis 2: Nonlocal correlation

For the second-order intensity correlation between detectors ${D}_{1}$(Alice) and ${D}_{2}$ (Bob), the coincidence measurements between Eqs. (9) and (10) are conducted by a gated heterodyne detection for the AOM-induced frequency-polarization correlated photon pairs, resulting in the selective choice of $\widehat{H}\widehat{V}$ product basis only:

19 $$\begin{aligned} \langle {R}_{Q1Q2}\left(0\right)\rangle &=\frac{{I}_{0}^{2}}{64}\left(\widehat{H}\cos\theta -i\widehat{V}\sin\theta \right)\left(\widehat{H}\cos\psi +i\widehat{V}\sin\psi \right)(cc)\\& =\frac{{I}_{0}^{2}}{64}\widehat{H}\widehat{V}{{{\sin}}}^{2}\left(\theta -\psi \right),\end{aligned}$$

where cc is a complex conjugate. For this gated heterodyne detection, the resolving time of a single photon detector must be much faster than the beating period to freeze the coincidence window^45^. Due to the action of AOMs resulting in the frequency-polarization correlation, i.e., $|\Psi \rangle ={|f\rangle }_{\mp }|H\rangle +{e}^{i\varphi }{|f\rangle }_{\pm }|V\rangle$, only $\widehat{H}\widehat{V}$ product is chosen for the DC-cut AC-pass filter, where Eq. (19) shows the inseparable intensity product of the quantum feature.

**Figure 4:**
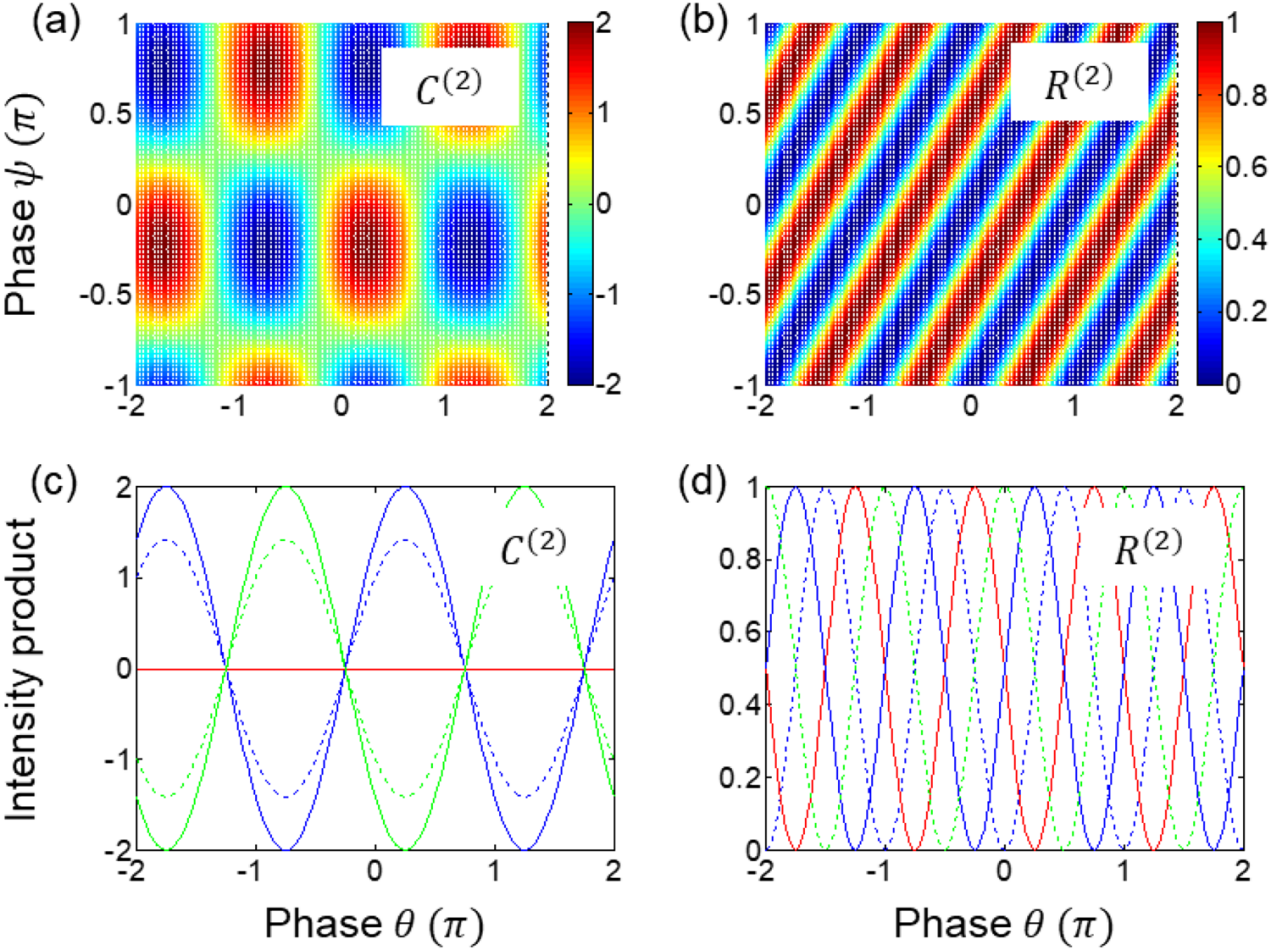
Numerical calculations of Eqs. (17) and (18). ${C}^{(2)}={C}_{1Q2Q}^{\xi =0}(0)$ in Eq. (17), ${R}^{(2)}={R}_{Q1Q2}(0)$. (bottom panels) $\uppsi =-\frac{\pi }{4}$ (Blue); 0(Dotted); $\frac{\pi }{4}$ (Red); $\frac{\pi }{2}$ (Dotted green); $\frac{3\pi }{4}$ (Green). The polarizer’s rotation angle $\uptheta (\uppsi )$ is for the path with (without) QWP in Fig. 1.

For this nonlocal quantum feature in Eq. (19), two independent polarizers $(\theta ;\psi )$ are considered for $\xi =0$ and $\varphi =\pi /2$ as a two-photon condition, satisfying local realism in both quantum erasers. Here, the polarizers act as a basis control as suggested by the Bell inequality test^41^, and demonstrated for the Bell inequality violation^47^. The same result of Eq. (19) is also obtained for the usual MZI case of Eqs. (5) and (6) for the MZI output port without QWP (see Section D of the Supplementary material). As analyzed for the Franson-type correlation^26^, thus, the selective measurement process is the key to the nonlocal correlation^5,28^. As shown in Section E of the Supplementary materials, however, no quantum feature exists between quantum erasers in blocks A and B, with and without QWP, i.e., between Eqs. (6) and (9) or Eqs. (4) and (10). Interestingly, thus, the phase relation between coincidently paired photons is quite important for the quantum feature (see lower left panel of Fig. 2) not only for the PBW-like super-resolution but also for the nonlocal correlation in Eq. (19) (discussed in Fig. 4). This also implies what the phase relation between higher-order entangled photons should be. In other words, Detectors 1 (2) and 3 (4) in Fig. 1 should be in the same party for the nonlocal correlation test, where the photons between them have a $\uppi /2$ phase shift in fringes, as shown in Fig. 2.

Figure 4 shows numerical calculations of both classical and quantum features derived in Eqs. (15), (16), and (19). Figure 4a is the direct intensity products between two output photons of Eqs. (15) and (16), whereas Fig. 4b is the result via a gated heterodyne detection for Eq. (19). Figure 4c and d are the corresponding details of Fig. 4a and b, respectively. The fringe shift of the product basis in Fig. 4d is the witness of the Bell inequality violation^47^. Due to the selective choice of the product bases in Eq. (19), the cost to pay for the quantum feature in Fig. 4b and d is 50% measurement event loss, as shown for the Franson-type nonlocal correlation^28^ and its applications^5^. Thus, the scheme of super-resolution in Fig. 1 satisfies the nonlocal quantum correlations if the AOM pair is used for the polarization-basis selective measurement via coincidence detection.

## Conclusion

Using a phase control of coherent photons in a modified quantum eraser scheme with QWP, a PBW-like super-resolution was analytically demonstrated for the fringe doubling and quadrupling effects, whose phase resolution is satisfied by the Heisenberg limit overcoming SQL. The role of QWP was to induce a $\uppi /2$ phase shift between orthogonal polarization bases of a single photon, resulting in fringe shifts between quantum erasers with and without QWP. Unlike coherently excited super-resolution^32,33^, the physics of the presented method was in the quantum eraser, where the QWP-induced phase shift was differently enacted to the polarizer of the quantum eraser, resulting in the $\pm\uppi /2$ fringe shifts from the non-QWP-based quantum eraser. Thus, the second (fourth)-order intensity correlation between them resulted in the fringe doubling (quadrupling) effect for the super-resolution. The analytically derived coherence solutions of the phase-controlled coherent photon-based super-resolution satisfied the Heisenberg limit in phase detection, overcoming SQL. For the nonlocal quantum correlation between the phase-controlled quantum erasers, a gated heterodyne detection was adopted for the polarization product-basis selection in coincidence measurements. For this, an AOM pair was used to induce frequency-polarization correlation between paired photons. Corresponding numerical calculations for both super-resolution and nonlocal quantum features were perfectly consistent with the conventionally observed results. Thus, the present study sheds light on a better understanding of quantum mechanics, where the phase relation between paired photons should play a key role in unveiling the quantum mystery.

## Methods

The MZI in Fig. 1 comprises a polarizing beam splitter (PBS) and a 50/50 nonpolarizing beam splitter (BS). The coherent photon is randomly generated from the attenuated laser L by Poisson statistics, whose mean photon number is set to be $\langle n\rangle <1$ to satisfy independent and incoherent photon statistics. For the higher-order intensity correlations between quantum erasers, bunched photons are randomly and statistically post-selected by a single-photon counting module^19^. The generation ratio of (N + 1)-bunched photons to N-bunched photons is determined by Poisson statistics at a few percent. For the particular order of intensity correlation, the coincidence measurements through the single-photon counting module play an essential role^19^. For random polarizations of an input photon, a $22.5^\circ$-rotated half-wave plate (HWP) is added before NMZI, where the laser L is vertically polarized. For the AOM-induced frequency-polarization correlation between path-paired photons, an oppositely diffracted photon pair is chosen for a common rf frequency. The polarizer’s angle is from the horizontal axis toward a counterclockwise direction^19^. For gated heterodyne detection, a DC-cut AC-pass filter is used to block the same polarization product bases of paired photons. Due to the random chance of transmission and reflection on either PBS or BS, all bunched photon cases in each BS are discarded for measurements of the second-order intensity correlation by the DC-cut AC-pass filter, resulting in the orthogonally polarized product-basis selection. Here, the terminology of ‘nonlocal’ is provided by the quantum eraser scheme, whose physical distance between the PBS and each polarizer is beyond the light cone, satisfying the violation condition of the cause-effect relation determined by the relativity theory.

### Supplementary Information

Supplementary Information.

## Data Availability

All data generated or analyzed during this study are included in this published article.
