# Supplementary material for: Phase-controlled coherent photons for the quantum correlations in a delayed-choice quantum eraser scheme
Source: Sci Rep. 2024 Jan 19;14:1752. doi: 10.1038/s41598-024-52125-0 (PMC10799079; doi:10.1038/s41598-024-52125-0)
Supplement: Supplementary file 1 — Supplementary Information. [file 41598_2024_52125_MOESM1_ESM.pdf]

Supplementary Materials

Phase-controlled coherent photons for the quantum correlations in a delayed-choice quantum eraser scheme by

Byoung S. Ham

Section A

Function of the polarizer

Regarding the role of a polarizer rotated at an angle θ from the horizontal axis as shown in Fig. S1(a), the resulting interference fringes is usually interpreted as polarization projections of the orthogonal polarization bases onto the θ axis: $\hat{H} \rightarrow \cos\theta\hat{p}$ and $\hat{V} \rightarrow \sin\theta\hat{p}$. In the viewpoint of quantum superposition between orthogonal bases, the original bases of $\hat{H}$ and $\hat{V}$ can be decomposed into two ad-hoc bases of $\hat{p}$ and its $90°$ rotated axis $\hat{\hat{p}}$. These two bases are of course mutually exclusive, resulting in no fringes to satisfy the particle nature of a photon. Along the new bases of $\hat{p}$ and $\hat{\hat{p}}$, thus, both $\hat{H}$ and $\hat{V}$ are now interfered, resulting in fringes. This is the quantum eraser, where along $\hat{\hat{p}}$, a fringe inversion results in, as in the MZI output ports:

$$I_p = I_0(1 + sin2\theta cos\varphi), \tag{SA-1}$$

$$I_{\bar{p}} = I_0(1 - sin2\theta cos\varphi). \tag{SA-2}$$

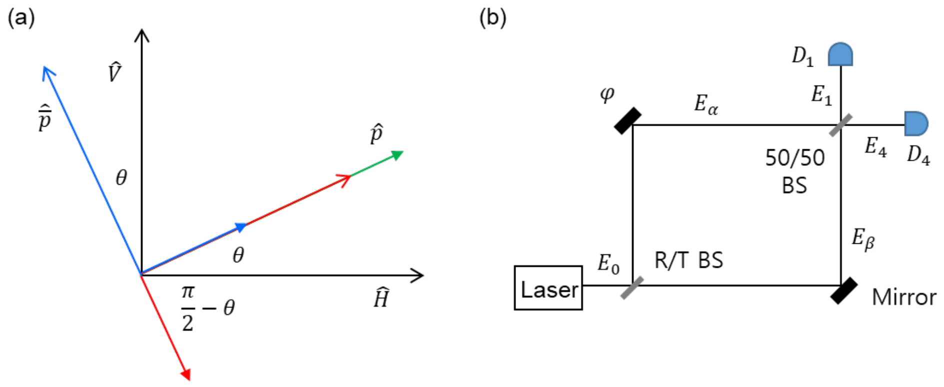

Fig. S1. An ad-hoc quantum superposition by the dynamic polarization axis given by a polarizer. R/T: reflection/transmission.

Equations (SA-1) and (SA-2) are for two quantum erasers observed in both NMZI output ports D_1 and D_4 (D'_4) without QWP in Fig. 1. Interestingly Eqs. (SA-1) and (SA-2) are exactly the same as the unbalanced MZI case for Fig. S1(b):

$$E_1 = E_0(cos\delta - sin\delta e^{i\varphi}), \tag{SA-3}$$

$$E_4 = E_0(cos\delta + sin\delta e^{i\varphi}), \tag{SA-4}$$

where $\cos\delta/\sin\delta$ represents the ratio of transmission to reflection by the adjustable R/T BS. Thus,

$$I_1 = I_0(1 - sin2\delta cos\varphi), \tag{SA-5}$$

$$I_4 = I_0(1 + sin2\delta cos\varphi), \tag{SA-6}$$

Compared to Fig. S1(a), the role of δ in Eqs. (SA-5) and (SA-6) is equivalent to θ in Eqs. (SA-1) and (SA-2).

Section B

Role of QWP for the PBW-like quantum feature

From Eqs. (7) and (8) in the main text without QWP, the intensity product between them is as follows for $\theta = \psi = \frac{\pi}{4}$:

$$\langle C_{14'}^{(2)}(0)\rangle = \langle C_{14}^{(2)}(0)\rangle = \langle C_{23'}^{(2)}(0)\rangle = \langle C_{23}^{(2)}(0)\rangle = \frac{I_0^2}{64} sin^2\varphi. \qquad \text{(SB-1)}$$

Similarly, $\langle C_{34}(0)\rangle = \langle C_{12}(0)\rangle$ results in, too. Thus, the four-photon intensity correlation is as follows for $\theta = \psi = \frac{\pi}{4}$:

$$\langle C_{1234}^{(4)}(0)\rangle = \frac{I_0^4}{256} sin^4\varphi, \qquad \text{(SB-2)}$$

where, the factor 2 is multiplied to each amplitude because of four photon interaction. Unlike QWP-based result in Eq. (18) in the main text, there is no fringe doubling occurs, except for the second-order intensity correlation in Eq, (SB-1).

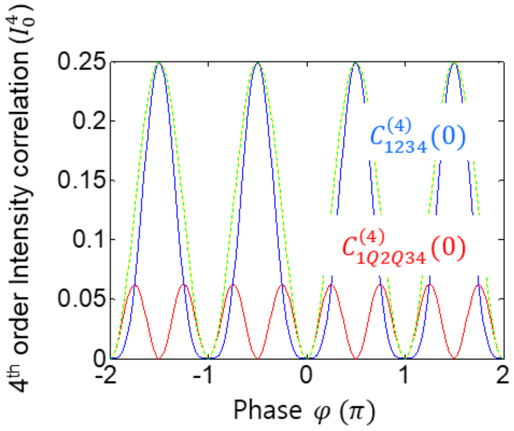

Fig. S2. Numerical calculations of fourth-order intensity correlations with (red) and without (blue) QWP. The greed dot curve is for Eq. (SB-1). The red curve is with QWP for Eq. (18).

Section C

Standard quantum limit without QWP

Figure S3 shows numerical calculations of the classical feature of the higher-order (N) intensity correlations for Fig. S3(a) between D_1~D_4. The MZI output intensities are $I_A(\varphi) = I_0(1 - \cos\varphi)$ and $I_B(\varphi) = I_0(1 + \cos\varphi)$. For the split case along the upper MZI output port, the followings are derived:

$$E_1(\varphi) = \frac{E_0}{4}(1 - e^{i\varphi}), \tag{SC-1}$$

$$E_2(\varphi) = \frac{iE_0}{4}(1 - e^{i\varphi}), \tag{SC-2}$$

$$E_3(\varphi) = \frac{-E_0}{4}(1 - e^{i\varphi}), \tag{SC-3}$$

$$E_4(\varphi) = \frac{iE_0}{4}(1 - e^{i\varphi}). \tag{SC-4}$$

The corresponding intensities are $I_1(\varphi) = I_2(\varphi) = I_3(\varphi) = I_4(\varphi) = \frac{I_0}{8}(1 - cos\varphi)$, resulting in the global-phase independence. For independent and individual events of $I_j(\varphi)$, the normalized higher-order intensity correlations are given by:

$$\langle C^{(N)} \rangle \propto (1 - cos\varphi)^N. \tag{SC-5}$$

Figures S3(b) and (c) are the numerical calculations of Eq. (SC-5) for N=1~4. As shown in Fig. S3(c), the resolution enhancement follows the standard quantum limit proportional to $\sqrt{N}$.

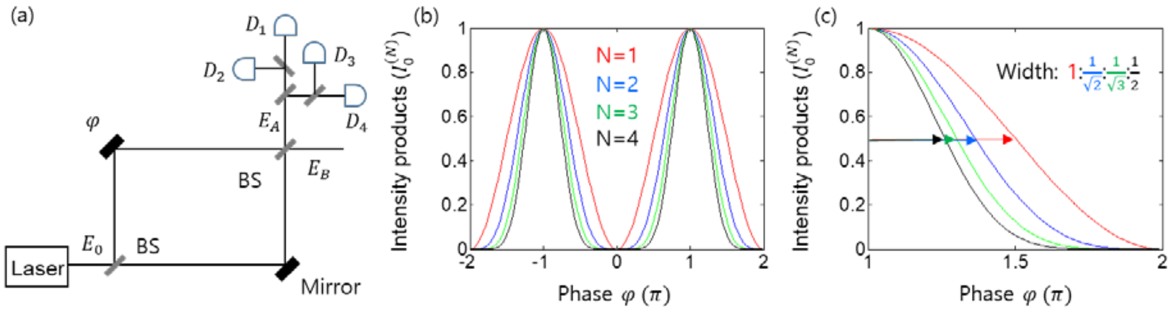

Fig. S3. Numerical calculations of the ordered intensities. BS: nonpolarizing 50/50 beam splitter.

Section D

Selective measurements

From Eqs. (5) and (6), the second-order intensity correlation R'_{14} is obtained as follows:

$$\mathrm{R}_{34}(0) = E_3 E_4(cc)$$

$$= \frac{I_0^2}{64}\left(\hat{H}cos\eta - \hat{V}sin\eta e^{i\varphi}\right)\left(\hat{H}cos\zeta + \hat{V}sin\zeta e^{i\varphi}\right)(\mathrm{cc})$$

$$= \frac{I_0^2}{64}\hat{H}\hat{V}(cos\eta sin\zeta - sin\eta cos\zeta)(\mathrm{cc})$$

$$= \frac{I_0^2}{64}\hat{H}\hat{V}\sin^2(\zeta - \eta), \tag{SD-1}$$

where a factor $\sqrt{2}$ is multiplied to Eqs. (5) and (6) for the two photon input. In Eq. (SD-1), a gated heterodyne detection makes the system selective for $\hat{H}\hat{V}$ product only, resulting in the nonlocal quantum feature.

Section E

Role of QWP for the nonlocal quantum feature

For the second-order intensity correlation between detectors D_1 (with QWP) and D_4 (without QWP), the coincidence detection between Eqs. (9) and (6) results in:

$$R_{1Q4}(0) = \frac{I_0^2}{64}\left(\hat{H}cos\theta - i\hat{V}sin\theta\right)\left(\hat{H}cos\zeta + \hat{V}sin\zeta\right)(cc)$$

$$= \frac{I_0^2}{16}\left(\hat{H}\hat{H}cos\theta cos\zeta - i\hat{V}\hat{V}sin\theta sin\zeta + \hat{H}\hat{V}(cos\theta sin\zeta - isin\theta cos\zeta)\right)(cc)$$

$$= \frac{I_0^2}{16}\{(cos\theta cos\zeta - isin\theta sin\zeta)(cos\theta cos\zeta + isin\theta sin\zeta) + (cos\theta sin\zeta - isin\theta cos\zeta)(cos\theta sin\zeta + isin\theta cos\zeta) + [(cos\theta cos\zeta - isin\theta sin\zeta)(cos\theta sin\zeta + isin\theta cos\zeta) + (cos\theta cos\zeta + isin\theta sin\zeta)(cos\theta sin\zeta - isin\theta cos\eta)]\}$$

$$= \frac{I_0^2}{16}[(cos^2\theta cos^2\zeta + sin^2\theta sin^2\zeta) + (cos^2\theta sin^2\zeta + sin^2\theta cos^2\zeta) + (cos^2\theta cos\zeta sin\zeta + sin^2\theta sin\zeta cos\zeta) + (cos^2\theta cos\zeta sin\zeta + sin^2\theta sin\zeta cos\zeta)],$$

$$= \frac{I_0^2}{16}(1 + 2sin\zeta cos\zeta), \qquad \text{(SE-1)}$$

where cc is complex conjugate. In Eq. (SE-1), two independent polarizers $(\theta;\zeta)$ are considered for $\xi = 0$ and $\varphi = \pi/2$ as a two-photon condition, satisfying local realism in both quantum erasers. For the coincidence detection, no AOM-induced effect is considered. In Fig. S4, no joint parameter relation is resulted between detectors with and without QWP. Thus, the phase relation between interacting photon pair is important compared to Eq. (19).

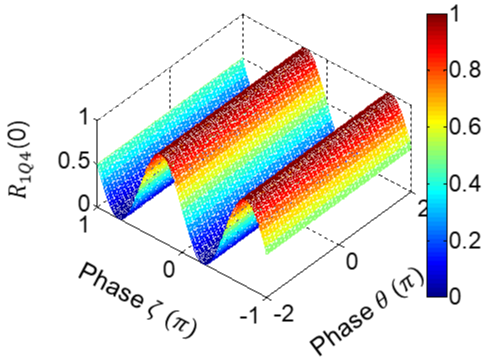

Fig. S4. Numerical simulations of Eq. (SE-1). $R_{1Q4}(0)$ is normalized.
